# Caries of Human Teeth

**Published:** 1883-11

**Authors:** 


### CARIES OF HUMAN TEETH.

Too late for insertion in this number has come to hand a valuable paper upon the above subject by Dr. Miller, of Berlin, which contains the results of his further investigations. Dr. Miller is an indefatigable student, and has the benefit of the best chemical laboratories of the world, together with the advice and co-operation of some of the great chemical teachers of Germany. His conclusions, therefore, are anxiously awaited, not alone by dentists, but by pathologists in other fields. The paper will be published in our next number.

